# A biodiversity hotspot losing its top predator: The challenge of jaguar conservation in the Atlantic Forest of South America

**DOI:** 10.1038/srep37147

**Published:** 2016-11-16

**Authors:** Agustin Paviolo, Carlos De Angelo, Katia M. P. M. B. Ferraz, Ronaldo G. Morato, Julia Martinez Pardo, Ana C. Srbek-Araujo, Beatriz de Mello Beisiegel, Fernando Lima, Denis Sana, Marina Xavier da Silva, Myriam C. Velázquez, Laury Cullen, Peter Crawshaw Jr, María Luisa S. P. Jorge, Pedro M. Galetti, Mario S. Di Bitetti, Rogerio Cunha de Paula, Eduardo Eizirik, T. Mitchell Aide, Paula Cruz, Miriam L. L. Perilli, Andiara S. M. C. Souza, Verónica Quiroga, Eduardo Nakano, Fredy Ramírez Pinto, Sixto Fernández, Sebastian Costa, Edsel A. Moraes Jr, Fernando Azevedo

**Affiliations:** 1 Instituto de Biología Subtropical, CONICET-Universidad Nacional de Misiones (UNaM), Bertoni 85, (N3370AIA) Puerto Iguazú, Misiones, Argentina; 2 Asociación Civil Centro de Investigaciones del Bosque Atlántico, Bertoni 85, (N3370AIA) Puerto Iguazú, Misiones, Argentina; 3 Departamento de Ciências Florestais, ESALQ, Universidade de São Paulo, Piracicaba, SP, Brazil; 4 Instituto Pró-Carnívoros Atibaia, Av. Horácio Neto, 1030 12954–010, Atibaia, SP, Brazil; 5 Centro Nacional de Pesquisa e Conservação de Mamíferos Carnívoros CENAP/ICMBio, Av. Hisaichi Takebayashi, 8600 12946–051, Atibaia, SP, Brazil; 6 Programa de Pós-graduação em Ecologia de Ecossistemas, Universidade Vila Velha (UVV), Vila Velha, ES, Brazil; 7 Instituto SerraDiCal de Pesquisa e Conservação, Belo Horizonte, MG, Brazil; 8 Floresta Nacional de Capão Bonito/ICMBio, Capão Bonito, SP, Brazil; 9 IPÊ - Instituto de Pesquisas Ecológicas, Nazaré Paulista, SP, Brazil; 10 Programa de Pós-graduação em Ecologia e Biodiversidade, Instituto de Biociências, Universidade Estadual Paulista – UNESP, Rio Claro, SP, Brazil; 11 PPG Ecologia- UFRGS, Porto Alegre, RS, Brazil; 12 Projeto Carnívoros do Iguaçu, Parque Nacional do Iguaçu/ICMBio, Rodovia BR 469 Km 22,5, Foz do Iguaçu, PR, Brazil; 13 Fundación Moisés Bertoni, Asunción, Paraguay; 14 Vanderbilt University, Nashville, TN, United States; 15 Departamento de Genética e Evolução, Universidade Federal de São Carlos, São Carlos, SP, Brazil; 16 Facultad de Ciencias Forestales, Universidad Nacional de Misiones (UNaM), Misiones, Argentina; 17 PUCRS, Faculdade de Biociências, Porto Alegre, RS, Brazil; 18 Department of Biology, University of Puerto Rico, San Juan, Puerto Rico; 19 Programa de Pós-graduação em Ecologia, Universidade Federal de Viçosa, MG, Brazil; 20 Instituto de Pesquisas Cananéia, Cananéia, SP, Brazil; 21 Instituto nacional de Medicina Tropical, Puerto Iguazú, Argentina; 22 Biotrópicos – Instituto de Pesquisa, MG, Brazil; 23 Departamento de Ciências Naturais - Universidade Federal de São João del Rei. São João Del Rei, MG, Brazil

## Abstract

The jaguar is the top predator of the Atlantic Forest (AF), which is a highly threatened biodiversity hotspot that occurs in Brazil, Paraguay and Argentina. By combining data sets from 14 research groups across the region, we determine the population status of the jaguar and propose a spatial prioritization for conservation actions. About 85% of the jaguar’s habitat in the AF has been lost and only 7% remains in good condition. Jaguars persist in around 2.8% of the region, and live in very low densities in most of the areas. The population of jaguars in the AF is probably lower than 300 individuals scattered in small sub-populations. We identified seven Jaguar Conservation Units (JCUs) and seven potential JCUs, and only three of these areas may have ≥50 individuals. A connectivity analysis shows that most of the JCUs are isolated. Habitat loss and fragmentation were the major causes for jaguar decline, but human induced mortality is the main threat for the remaining population. We classified areas according to their contribution to jaguar conservation and we recommend management actions for each of them. The methodology in this study could be used for conservation planning of other carnivore species.

Apex predators, particularly large carnivores, are key components of ecosystems as they help maintain biodiversity and ecological processes via multiple food web pathways^1^,^2^. These species require large areas of habitat with a stable prey base for their long-term survival, and they are particularly susceptible to population declines in human modified landscapes^3^. Human persecution, global habitat loss and fragmentation have exposed most species of large carnivores to extinction risk worldwide^2^. However, the impact of these threats varies across regions and species^2^. While some populations of large carnivores in North America and Europe are recovering as forested areas increase, along with protective legislation and greater human tolerance^4^,^5^, most large tropical carnivore populations are still declining^2^,^6^.

Tropical forests sustain most of the global terrestrial biodiversity, but they have suffered high rates of deforestation and defaunation of large vertebrates^7^,^8^. Even though, the loss of top predators in these forests can have direct effects on the diversity and function of these biologically diverse ecosystems^7^,^2^. The conservation of top predators often is a challenge that requires large efforts to evaluate population status through extensive areas, and coordinated international efforts to develop conservation planning strategies^2^,^6^.

The Atlantic Forest (AF) of South America is one of the Earth’s Biodiversity Hotspots with high levels of diversity and endemism^9^.The AF extends through more than 1.7 million km^2^ across Brazil, Argentina and Paraguay, and during the last centuries, it has undergone an intense landscape transformation. Today less than 12% of the original forest cover still exists^10^. Due to the high levels of diversity and the extreme loss of forest cover, the region is among the eight “hottest hotspot” of the world^9^ and is considered a top priority area by most of the largest international NGOs (e.g. Word Wildlife Fund, Conservation International). Most of the remaining AF has lost its largest mammals, including its top predator, the jaguar (*Panthera onca*; refs 11 and 12). If this trend continues, the AF will be the first tropical forest ecoregion to lose its top predator^13^.

The jaguar is the top predator of the Neotropical region, currently occurring from southern United States to northern Argentina. It has disappeared from about 54% of its original range, due to habitat loss, depletion of the prey base and human persecution^14^. Although it is listed as near threatened by the IUCN^6^, it has become locally extinct or critically endangered in some areas, including the AF, which is currently the southern distribution limit of the species^14^,^15^,^16^.

Historically, the jaguar occurred throughout the AF^14^,^16^, but its’ current distribution has been greatly reduced. Although many research teams have evaluated the population status of the jaguar in different regions or countries within the AF^15^,^16^,^17^,^18^,^19^,^20^,^21^, no study has evaluated its’ status across the entire ecoregion. During the last 10 years, governments, scientists and NGOs developed conservation plans for the Argentinean AF^22^ and the Brazilian AF^23^, but there is still no international conservation strategy for the entire region. Saving the jaguar population in the AF requires a better understanding of its status throughout the entire region, as well as international planning and cooperation for conservation^24^. This article addresses this limitation by combining data sets from 14 jaguar research projects from across the AF to: (i) evaluate current jaguar habitat availability, (ii) estimate the area of occupancy and population size, (iii) identify potential connectivity cost among subpopulations, (iv) identify the main threats to jaguar conservation in the region, (v) propose integrated actions for long-term conservation, and (vi) use our study as a model to assist conservation efforts of jaguars and other large carnivores in other regions facing similar conservation problems.

## Results

### Habitat suitability for jaguars and areas of occupancy

**Figure 1:**
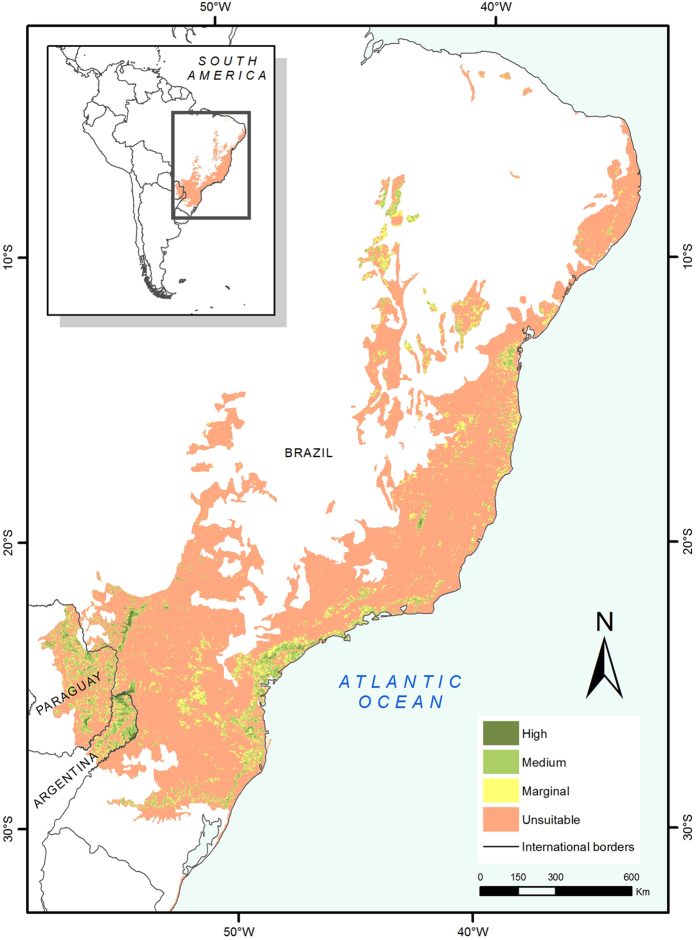
Habitat suitability for jaguars in the Atlantic Forest. The colored area inside the left corner inset details the location of the study area in South America. The map was created with ArcGis 10.3 (www.arcgis.com).

Of the original 1.7 million km^2^ of the AF, 15.1% was classified as habitat currently suitable for jaguars, but only 0.7% (9,017 km^2^) occurred in areas classified as highly suitable (Fig. 1). The remainder of suitable habitat was classified as medium suitability (6%; 81,473 km^2^), or marginal suitability (8.4%; 114,860 km^2^). Jaguar habitat loss varied among countries: Brazil lost 87% of its’ original AF suitable habitat, Paraguay 64% and Argentina 39%. Of the total remaining jaguar suitable habitat in the AF, 27% is fragmented into 12,608 patches smaller than 100 km^2^, 43% into 305 fragments of between 100 km^2^ and 1,000 km^2^, and 29% into 35 patches larger than 1,000 km^2^.

The habitat suitability model for jaguar that we developed for these estimations was highly significant (p < 0.001, area under the receiver operating characteristic: AUC = 0.82 ± 0.05) and presented low omission error (~22%). The post-hoc validation using the independent recent presence-only records confirmed that the model was highly accurate, with only 5.1% omission error. According to our model, jaguar habitat in the AF was mainly determined by high forest cover and marshlands (53.1%), intermediate elevation (19.4%) and low human accessibility (17.3%).

**Figure 2:**
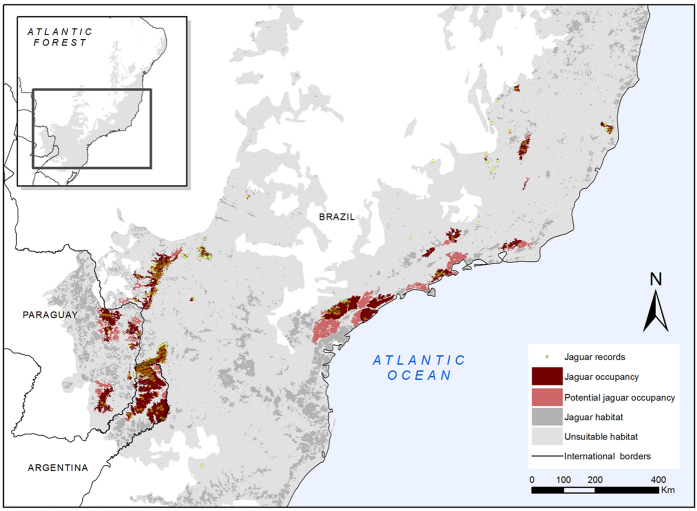
Jaguar records obtained along the AF and the areas of jaguar occupancy (AJO), the areas of potential jaguar occupancy (APJO) and the distribution of available jaguar habitat in the Atlantic Forest. The map was created with ArcGis 10.3 (www.arcgis.com).

Jaguar presence was confirmed in only a few areas of the AF (Fig. 2). In the coastal region of Brazil, the species is apparently extinct in the southern and northern extremes, with populations currently confined to the states of São Paulo, Rio de Janeiro and Espírito Santo. In the interior portion of the AF, the species occurs in the state of Minas Gerais and along the Paraná River basin, in habitat fragments in Argentina, Paraguay and Brazil (Fig. 2). We estimated that the area of jaguar occupancy (AJO) in the AF covers only 35,441 km^2^. If we include as AJO the areas with jaguar records nearby (closer than 1.7 km), the AJO increases to 37,825 km^2^. Thus, the jaguar is occupying only 2.8% of the AF and 18.4% of the current jaguar suitable habitat. In addition, 16,420 km^2^ of jaguar habitat (1.2% of the AF and 8% of the total current jaguar suitable habitat) were very close to AJOs and are areas of potential jaguar occupancy (APJO, Fig. 2). In 151,105 km^2^ (73.6%) of available jaguar habitat in the AF we do not have jaguar records, most probably representing areas without jaguars (Fig. 2).

The AJOs were mainly the largest patches of remaining habitat. Jaguars appear to have disappeared from 96% of the habitat fragments with less than 100 km^2^, 86% of the habitat fragments between 100 km^2^ and 1,000 km^2^, and 40% of the habitat fragments larger than 1,000 km^2^.

### Jaguar density and population size

Jaguars were not detected in eight of the 30 camera-trap surveys conducted in the AF (Table 1). These surveys without jaguar records were conducted in the AJO of the Serra do Mar, Serra do Mar do Norte and Serra dos Órgãos (Supplementary Fig. S1 and Table S1). In 13 other surveys, the number of individuals recorded was insufficient to apply spatially explicit capture-recapture population models (SECR) to estimate jaguar density (Table 1). We estimated densities based on data from nine surveys from six areas. In these surveys, we recorded between eight and 42 individuals and estimated densities between 0.66 and 2.42 jaguars/100 km^2^ (Table 1). The highest density estimates were obtained in the Linhares-Sooretama region and in the Morro do Diabo State Park, and the lowest were from a forest block including the Intervales State Park and Alto Ribeira State Touristic Park (Table 1, see also Supplementary Fig. S1).

The estimated number of jaguars for each region varied according to density estimates and the size of habitat fragments that were surveyed (Table 2). The largest subpopulations occurred in the Green Corridor, the Upper Parana-Paranapanema and the Serra do Mar region (Table 2). Smaller subpopulations, were located in the Mbaracayú and Linhares-Sooretama regions (Table 2).

### Priority areas for jaguar conservation

**Figure 3:**
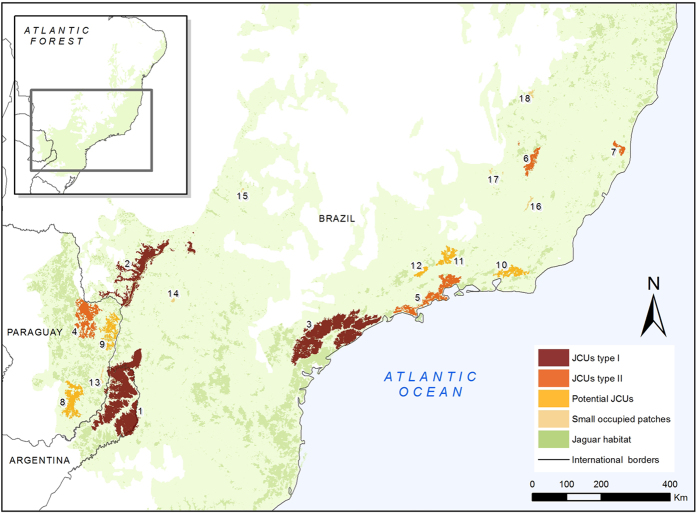
Distribution of the Jaguar Conservation Units (JCU), the Potential Jaguar Conservation Units (PJCU) and the small fragments with jaguar presence. References: (1) Green Corridor, (2) Upper Parana-Paranapanema, (3) Serra do Mar, (4) Mbaracayú, (5) Serra do Mar Norte, (6) Rio Doce, (7) Linhares-Sooretama, (8) San Rafael, (9) Itaipú, (10) Serra dos Órgãos, (11) Itatiaia, (12) Campos do Jordao, (13) East Paraguay, (14) Das Perobas Reserve, (15) Rio Tiete-West SP, (16) PE Serra do Brigadeiro, (17) Mantiqueira and (18) Espinhaço. The map was created with ArcGis 10.3 (www.arcgis.com).

We identified seven areas with presence of males and females that were categorized as Jaguar Conservation Units (JCU) and five Potential Jaguar Conservation Units (PJCU; no detection of both sexes). Three JCUs contained, or probably contained 50 individuals or more and were categorized as JCU Type I (JCUs with higher probability of long-term population persistence): the Green Corridor, the Upper Parana-Paranapanema, and the Serra do Mar regions (Table 2, Fig. 3). These three JCU were all larger than 5,000 km^2^. While the population of the Serra do Mar JCU was probably smaller than 50 individuals, we classified it as a Type I JCU because the habitat availability of the area is large enough to maintain a larger population (Table 2). These three JCUs together constitute more than 60% of the current priority areas for jaguar conservation in the AF (Table 2).

The other four JCU are smaller and probably contain less than 50 individuals each consequently were classified as JCU Type II. These JCUs varied in terms of size, density and habitat conditions (Table 2). The largest is Mbaracayú, in eastern Paraguay, which encompasses 4,086 km^2^ of jaguar habitat. The other three vary between 503 km^2^ and 3,915 km^2^ and are located in Minas Gerais State and the coastal region of Brazil (Fig. 3). These Type II JCUs cover 18% of the priority areas for jaguar conservation in the AF (Table 2).

We identified five PJCUs in western Paraguay and the Brazilian coast (Fig. 3). The size of the PJCUs varied between 539 km^2^ and 2,941 km^2^, and together they constitute 16% of the priority area for jaguar conservation in the AF (Table 2). Despite the presence of jaguars in these PJCUs, jaguar records were scarce and densities are apparently very low (Table 1). Small fragments with jaguar presence were spread across different areas of the AF and constitute a very small fraction of the jaguar habitat (Table 2). Records in these areas are occasional and probably of nonresident individuals.

**Figure 4:**
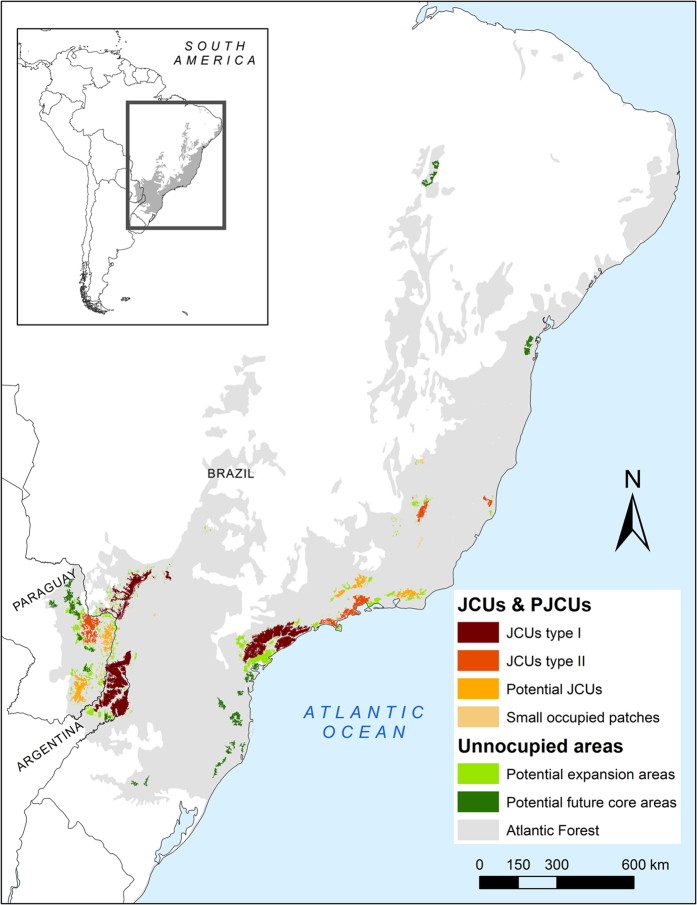
Other important areas for jaguar conservation in the Atlantic Forest. Potential core areas may be considered as potential areas to reintroduce the species to create new subpopulations. Potential expansion areas may be considered to enlarge JCU, PJCU and Potential core areas. The map was created with ArcGis 10.3 (www.arcgis.com).

Several areas where jaguar were not detected could be important for jaguar conservation in the future because they are large areas with good quality jaguar habitat. Two of these areas are located near the Green Corridor JCU and the Serra do Mar JCU, and if they were connected they could expand the size of these existing JCUs (Fig. 4). We also identified potential core areas that may sustain jaguar’s subpopulations in the future, and are potential areas for reintroduction programs. These areas are located in the northern part of the AF (Bahia and Piauí states of Brazil), along the coast of Paraná and Santa Catarina states (Brazil), and in western Paraguay (Fig. 4). These areas varied between 232 and 1,072 km^2^ and together cover 12,218 km^2^ of potential core areas.

### Potential connectivity among the Jaguar Conservation Units

The relative cost (i.e., costs for a jaguar attempting to move between two JCUs) or potential connectivity among the JCUs was highly variable. The Linhares-Sooretama and Rio Doce JCUs showed the highest connectivity cost and isolation of all the JCUs (Supplementary Table S2). Other JCUs and PJCUs could be grouped into two main regions: the Upper Paraná Atlantic Forest-JCUs, in the West, and the Coastal Atlantic Forest-JCUs, in the East (Supplementary Table S2). Connectivity between these two regions appears to have excessively high cost to allow jaguar movements (Supplementary Table S2). Furthermore, the Upper Paraná Atlantic Forest-JCUs showed higher cost distance values, suggesting more isolation, whereas the Coastal Atlantic Forest-JCUs present lower values and thus higher potential of connectivity (Supplementary Table S2).

### Jaguar threats

The relative importance of threats varied among the different JCUs (Table 2). Ranked in order of importance, the threats included poaching, scarcity of prey, small population size, site isolation, human retaliation due to cattle predation, habitat loss, and road kills.

## Discussion

The population status of the jaguar in the AF is critical. Habitat loss and fragmentation have had a tremendous impact, and the species is locally extinct in most of the region. The few remaining subpopulations are small, scattered, highly isolated, and associated with relatively large forest remnants. This dramatic situation is similar to that faced by endangered large carnivore species in other regions of the World^2^.

Considering that a couple of centuries ago the species inhabited all the AF^14^,^16^, we found that approximately 85% of the jaguar habitat has been lost, and less than 7% of the region has medium to highly suitable habitat. Clearly, habitat loss has been the major driver of jaguar population decline in the AF, as was previously described for regions within the AF^11^,^12^,^16^,^25^. Our model shows that loss of forest cover and marshlands, and increases in human accessibility had a negative effect on jaguar habitat suitability in the AF. These changes were related to demographic and economic processes that affected Brazil, Paraguay and Argentina at different times and different degrees^26^. Most of the jaguar habitat in the Brazilian AF was probably lost between 1900 and 1980 due to the development of cities and large-scale agriculture^10^. In Paraguay, the loss of jaguar habitat mostly occurred during the last 30 years with the expansion of large-scale agriculture^27^. In Argentina, there has been less deforestation and degradation, and a larger proportion of the original habitat remains^26^.

We estimate that less than 27% of the suitable jaguar habitat of the AF contains jaguars. Although the species occurs in some relatively small and medium-size fragments, jaguars are mainly present in fragments larger than 1,000 km^2^. Habitat fragmentation can produce a large impact on the persistence of carnivores^3^,^28^,^29^. Only large areas of suitable habitat can sustain jaguar populations that are resilient to stochastic events^29^. According to our results, more than 70% of the habitat in the AF is fragmented into small remnants that cannot sustain more than 10 individuals at current densities. Jaguar subpopulations in these small fragments have a high probability of becoming extinct in a short period of time^29^,^30^, and have already been shown to lose genetic diversity at a very high rate^31^. Therefore, it is probable that habitat loss and fragmentation were, in an interaction with poaching and scarcity of prey, the main causes of local jaguar extinctions and large areas of empty suitable habitat.

The absence of records and the low rate of jaguar pictures in many camera-trap surveys of the AF suggest that several remnant subpopulations occur at very low densities. This is worrying given that most of these surveys were conducted in large forest areas of good jaguar habitat (e.g. protected areas), suggesting that population decline and local extinction are not only occurring in small fragments because habitat loss and fragmentation, but also in larger and more connected habitat fragments. The few areas where we recorded several individuals and could estimate densities, in general, were well-managed protected areas with relatively low poaching pressure^20^,^21^.

Poaching can reduce jaguar densities in the AF even in areas of good habitat quality^20^. Illegal hunting is widespread in the AF and is responsible for the frequent poaching of jaguars^20^,^32^ and the depletion of their prey base^33^,^34^,^35^,^36^. Jaguar killing in the AF is frequent, mainly by poachers that consider the species a trophy or by cattle ranchers that eliminate individuals as retaliation for livestock losses^20^,^32^,^37^. Large home range requirements of carnivores often expose them to the edges of protected areas, where they are in contact with human activities and are susceptible to high levels of human-induced mortality^3^. This “edge effect” could drive important changes in abundance inside the protected areas and increase the effect of habitat fragmentation^3^,^38^,^39^. Jaguar roadkill is also a threat in the AF, where the protected areas are adjacent to or crossed by roads or highways^40^. Road-killed jaguars have been recorded in protected areas of the AF, and thus, roads emerge as an additional threat for the species^40^.

**Figure 5:**
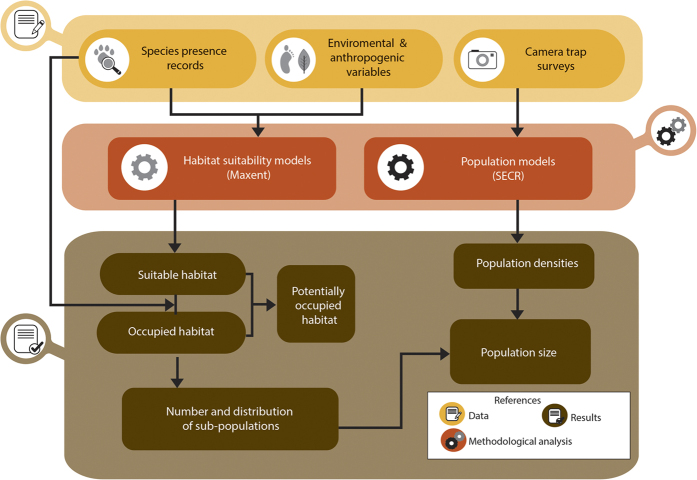
Methodology used to estimate the distribution and size of the subpopulations and the population size of jaguars in the Atlantic Forest. The same methodology could be used to evaluate the populations status of other carnivore species. The authors created this figure.

The jaguar population of the AF is small and severely fragmented into a few subpopulations that we defined as Jaguar Conservation Units. As we have not completely surveyed any of the JCU, we do not have the exact population size of them. Considering that most of the camera-traps surveys were conducted in areas with high habitat quality within protected areas^20^,^21^, it is highly probable that the density in the other areas are much lower. The low rate of jaguar records (track, feces, sightings, etc.) found in these areas in our and other studies^15^,^41^ support this hypothesis. Considering this and the extent of the AJO, it is probably that all the JCUs Type II have less than 25 animals each, that none of the JCU Type I have more than 85 individuals, and that the total population of the AF is roughly estimated to be in the range of between 150 and 300 individuals (Table 2, Fig. 5).

**Figure 6:**
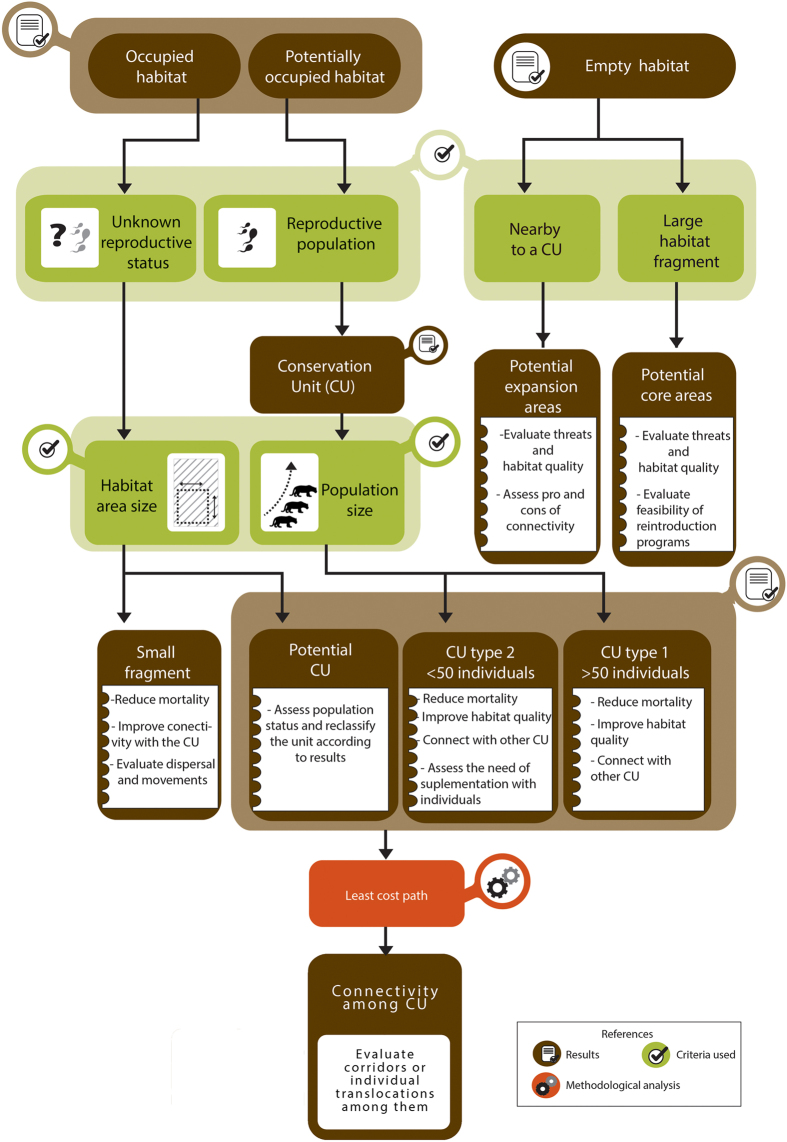
Methodology used to define the important areas for conservation of the species and management recommendations for each area. The same methodology could be used for conservation planning of other carnivore species. The authors created this figure.

Previous population viability analysis of jaguars estimate that populations with 50 individuals may persist in the medium-long term (100 years) if mortality is low^30^,^42^, while populations with fewer than 25 individuals have low probabilities of surviving for 100 years^30^,^42^. However, if the carrying capacity of a population is relatively high (e.g. large protected habitat with high prey populations), the chances of surviving increase considerably^30^,^42^. If we consider our highest density estimates as the potential carrying capacity for the AF, most of the JCUs are far below their carrying capacity. These subpopulations have the potential to increase in size and viability if protection is enforced and their numbers and those of their prey are allowed to increase. Currently, their small population size and isolation from other fragments have already resulted in loss of genetic diversity and likely decreased evolutionary potential^31^,^43^, implying that additional measures (*e.g.* restoration of connectivity to other areas or supplementation of individuals) will be needed for long-term persistence (Table 2, Fig. 6).

In contrast to other large carnivores with higher reproductive potential (e.g. leopards, pumas, tigers), jaguar populations cannot support even intermediate levels of harvest, and they decline rapidly when mortality is high^42^,^29^. In this scenario, reducing jaguar mortality appears to be the most important action to conserve jaguars in AF (Table 2, Fig. 6). Poaching must be reduced by increasing support for law enforcement, but also by developing high-impact education and communication campaigns^20^, as well as by implementing sustainable alternatives of living for communities neighboring protected areas. Reducing illegal hunting will not only reduce jaguar killing, but it will also increase its’ prey base and the carrying capacity of forest fragments. In addition, the loss of jaguars in retaliation to predation on livestock must be reduced through proactive policies from governmental agencies. For the smallest populations, periodic arrivals or supplementation of individuals could increase the probability of persistence^30^, and this must be considered as a viable alternative if we want these populations to persist in the long term (Table 2, Fig. 6).

Enhancing landscape connectivity is a key strategy of modern biodiversity conservation worldwide^44^. Our results show that the potential connectivity between JCUs is highly variable. The high cost and long distances between the JCUs in the Upper Parana Atlantic Forest will be a challenge for creating successful corridors. In this region, the role of the Itaipu PJCU is crucial, acting as a potential stepping-stone between the Green Corridor, the Upper Parana-Paranapanema and Mbaracayú JCUs (Fig. 3 and Supplementary Table S2). These last two JCUs could also play a very important role as a connection between the Atlantic Forest and Pantanal jaguar populations^44^. The Coastal Atlantic Forest JCUs show higher potential for connection. Hence, it will be more effective to promote corridors as a management policy in this area. In contrast, the high cost distance value of Rio Doce and Linhares-Sooretama JCUs suggests that they are highly isolated from the rest of the JCUs and that the chances of arrival of jaguars via natural dispersal will be extremely low. Furthermore, it seems quite difficult to implement natural functional corridors between the Upper Parana Atlantic Forest JCUs and Coastal Atlantic Forest JCUs since the cost-distance value is very high. In this scenario, we will need to develop an active management strategy including carefully planned and monitored translocation or supplementation of individuals to reinforce some jaguar populations and maintain their genetic diversity (Table 2 and ref. 43).

This study is the results of a collaborative effort among scientist of different countries to save the top predator of the AF. By joining sparse data and using different analytical techniques, we evaluated the size and distribution of AF´s jaguar subpopulations (Fig. 5). Using a combination of new and previous^14^,^45^ approaches, we defined the priority areas to conserve the species and proposed spatially explicit conservation actions (Fig. 6). This step-by-step process was an efficient way to transform basic information into management recommendations, and could be applied to jaguar populations in other regions, or other species of large carnivores.

Our work can be considered as the first step to understand the general population status of jaguars in the whole AF and guide priority conservation actions. However, this effort must be continued to improve our knowledge, cover existing information gaps and refine the conservation strategies. In the near future, population surveys in the AF should focus on exploring areas where jaguar presence was not confirmed (APJO and large high quality habitat patches identified by our model), regularly monitoring jaguar population size of the JCUs (including the evaluation of unsurveyed areas of the JCU) and its genetic diversity. In addition, it is crucial to understand how the jaguars move in this fragmented landscape, evaluating the feasibility and effectiveness of the establishment of corridors and techniques for supplementation or translocation of individuals (Table 2). Finally, it is also necessary to evaluate the best ways to reduce the human induced mortality of jaguars by analyzing alternatives to solve jaguar-cattle rancher conflicts, and to reduce poaching and the impact of roads.

The extinction of jaguars in the AF could have important consequences^13^. In the absence of jaguars, it is expected that populations of other species and many processes will change with unpredictable consequences for the ecosystem^1^,^2^. To protect and increase the jaguar populations and the natural areas that they depend on, constitute an enormous challenge for the next decades, but there are reasons for hope. Our population estimates for the Green Corridor (Table 1) suggest that this subpopulation is increasing after a marked decline in the 1990’s^20^, probably as a result of efforts of governmental institutions and NGOs in combating poaching and other threats. The survivorship of small and presumably isolated subpopulations in the Morro do Diabo and Linhares-Sooretama areas during the last decades also brings hope, in spite of their documented loss of genetic diversity^31^,^40^. Finally, the increasing collaboration among institutions of Brazil, Paraguay and Argentina to coordinate and develop transnational actions to study, monitor and conserve the species is also an asset.

During the last decades, a large amount of scientific evidence demonstrated the importance of large carnivores as key parts of ecosystems^1^,^2^. However, the survival of these species is still a challenge, especially in tropical ecosystems. Following the creation of the Large Carnivore Initiative for Europe Specialist Group for the IUCN, Ripple *et al*.^2^ proposed the creation of a Global Large Carnivore Initiative to maintain and restore, in coexistence with people, viable populations of large carnivores as an integral part of ecosystems and landscapes. We agree with this vision, and propose to put special attention and effort in the most threatened regions and ecosystems of the World. In this context, special attention must be given to the continued challenge of conserving the jaguar population of the Atlantic Forest.

## Methods

### Habitat suitability for jaguars

To evaluate the size and location of the remaining suitable areas for jaguars in the AF, we developed a species distribution model. We gathered 2,179 jaguar presence points (Fig. 2) collected in the region between 2003 and 2014 by 14 teams researchers and its collaborators involving more than 300 hundred people. Jaguar records correspond to camera-trap pictures, locations of collared individuals, poached or road-killed animals, sightings and jaguar confirmed tracks and feces obtained in systematic and non-systematic surveys (Supplementary material Methods S1). To reduce the spatial correlation among records from this dataset (e.g. many records corresponding to the same individual), we divided the study area in cells of the size of a female jaguar potential home range in the AF (144 km^2^), the smallest range of an individual of the species in region^11^, and randomly selected only one presence point from each cell^11^,^16^,^46^. The resulting 72 presence records that remained were partitioned randomly into training (70%) and testing (30%) datasets for cross-validation with replacement (n = 10).

We selected six non-correlated (Pearson’s r < 0.70) environmental and anthropogenic variables as predictors, from an initial set of 18 variables (Supplementary Table S3) that explained jaguar distribution in previous studies performed in the region^11^,^12^,^16^,^25^. The final variables were: human accessibility cost, % of natural habitats (native forest cover and marshlands), % of pastures, human population density, distance to rivers, and elevation. Because the characteristics of the species and the methodologies used in our surveys prevented obtaining locations of true absences, we chose the Maxent algorithm (Maxent 3.3.3 k) for running species distribution models^47^,^48^. As model parameters, we used a convergence threshold of 10−5 with 500 iterations, 10,000 background points, auto features, random seed, analysis of variable importance and response curves^48^.

The logistic output resulted in an average model with values ranging from 0 (unsuitable) to 1 (suitable)^47^. We set the ‘maximum test sensitivity plus specificity’ (0.364) as a decision threshold rule^49^ making the distinction between suitable (≥0.364) and unsuitable (≤0.364) areas. Then, we reclassified the final model in four classes with equal intervals, but adjusting the first value 0.250 to the threshold of 0.364. Final models resulted in: unsuitable (0 to 0.364), and the suitable habitat divided in marginal (0.364 to 0.5), medium (0.5 to 0.75) and highly suitable (0.75 to 1) areas for jaguars.

We evaluated the final model by the area under the receiver operating characteristic curve (AUC) value, a threshold-independent measure of overall model performance (mean ± standard deviation; ref. 50). The AUC ranges from 0 to 1, assuming that AUC ≥ 0.75 is a high score^51^. We also evaluated omission errors and model significance by binomial probability associated to the threshold used^52^. Finally, after the conclusion of the modeling analysis, we gathered new records from the field (n = 107, Supplementary material Methods S1), which we decide to use as an independent dataset to further test the model’s predictive ability for jaguar occurrence in the AF.

### Estimation of areas of jaguar occupancy

To identify the fragments of habitat with jaguar occupancy along the AF (as used by the IUCN to assess species status), suitable habitat areas obtained in the final model were converted to polygons, splitting the resulting habitat fragments by main and secondary roads. We overlaid all the jaguar occurrence points onto suitable habitat remnants, selecting those with confirmed jaguar presence. As some occurrence points were located outside the suitable habitat fragments, we calculated the median distance from these points to the closest habitat fragment (1.7 km). This value can be interpreted as the distance that jaguars usually reach when they move outside the suitable habitat. Therefore, to be conservative, we only considered as area of jaguar occupancy (AJO) every fragment of continuous suitable habitat containing jaguar records and those that had a jaguar record closer than 1.7 km (Fig. 5). Additionally, we considered as areas of potential jaguar occupancy (APJO) those fragments without jaguar records inside or near them, but that were closer than 1.7 km to an occupied fragment of suitable habitat (Fig. 5).

### Estimation of jaguar abundance, density and population size

To estimate relative abundance and density, we compiled jaguar records from 30 camera-trap surveys conducted between 2003 and 2014 (Table 1, see also Supplementary Fig. S1 and Table S1). Most of the surveys were specifically designed to evaluate jaguar abundance and were performed by our own teams, but we also included information from surveys conducted by other collaborators (17% of the surveys). The surveys covered portions of most of the largest fragments of remaining jaguar habitat in the AF, including areas with different levels of human activity and legal protection (Supplementary Fig. S1 and Table S1).

The surveys varied widely in terms of effort, distance among cameras-trap stations and area covered, and jointly they accumulated more than 80,000 camera-trap days from more than 900 different stations located in jaguar suitable habitat (Table 1, Supplementary Table S1). In general, camera-traps were deployed in pairs, facing each other along roads, trails or inside the forest, trying to cover places regularly used by jaguars. We used photographs to identify individual jaguars through their unique spotting pattern, and the sex and age by the presence or absence of scrotum and corporal build. For density estimation we only used data of perceived adult animals. Surveys with relatively few jaguar records (<6 individuals) were only used to estimate jaguar photographic rate and to confirm the presence of females in the area.

We estimated jaguar densities through spatially explicit capture-recapture population models (SECR) that combine capture-recapture records with their geographic location^53^,^54^. These models have been used previously to estimate jaguar density^55^,^56^,^57^, and simulations studies show that they generate the less biased density estimations in a wide range of conditions in relation to different sizes of areas covered by cameras and jaguar home range sizes^57^. To apply the SECR models, we used the Bayesian approach and Markov chain Monte Carlo simulations through the R package SPACECAP 1.1.0^54^,^58^,^59^. A detailed description of the procedures and parameters employed to estimate density is provided in Supplementary material Methods S2.

The lack of records of jaguar individuals at different stations in the Mbaracayu survey precluded the use of SECR models in this area. We thus estimated its jaguar density using non-spatial capture-recapture models in combination with information of the estimated home range size of the animals^20^,^60^ and using the jackknife estimator of abundance in the program CAPTURE2^61^. The effectively sampled area was estimated by applying to every camera-trap station a buffer equal to the radius of the mean home range of three animals monitored by GPS collars in this area (5.8 km; Ramirez *et al*., unpublished results). The resultant polygons around each camera were merged into one polygon that was considered the sampled area^20^,^55^.

To obtain the population size, in areas where we obtained density estimates with camera traps and population models, we used the NSuper parameter obtained in SECR analysis as the number of animals present in the surveyed area (Fig. 5). We also used 95% confidence interval limits as the minimum and maximum number of individuals living in each area.

### Identifying priority areas for jaguar conservation

We identified priority areas for jaguar conservation, defined as Jaguar Conservation Units, as those containing a jaguar population and suitable habitat. This approach was originally proposed by Sanderson *et al*.^14^, and has been used widely to update and redefine the JCUs originally proposed (e.g., refs 11,45, 62 and 63). In our work, we defined JCUs as those habitat units with confirmed presence of females and males as a proxy for existing reproductive populations (Table 1 and Fig. 6). We classified these JCUs into two categories, according to the known number of adult individuals present: Type I JCUs were areas with an estimated population size of ≥50 adults and Type II JCUs were areas with <50 adults (Fig. 6 and ref. 14).

To identify habitat units that can potentially constitute JCUs, we focused on the areas of jaguar occupancy and potential occupancy, and grouped them into those that were less than 15 km from a fragment with jaguar presence. This distance is the radius of the largest home range estimated for jaguar in the AF (Morato *et al*. unpublished results), and could be considered as a distance that is not usually traveled by resident jaguar individuals outside suitable habitat. One exception in this grouping procedure was Morro do Diabo State Park (Brazil) that was included in the Upper Parana-Paranapanema JCU. This fragment was more than 15 km apart from the other fragments of this Unit, but historical and political issues determine the feasibility of the development of a common conservation strategy and management actions with the rest of this JCU.

In this contribution, we also classified other habitat units into different categories according to their importance for jaguar conservation (Fig. 6). Habitat units occupied by jaguars but without the confirmation of males and females were classified according to their potential of becoming a JCU. Areas that had habitat in good condition (medium to high suitability) larger than the habitat in good condition of the smallest JCU (230 km^2^ in Linhares-Sooretama JCU) were categorized as ‘Potential Jaguar Conservation Units’ (PJCU, 42). Areas with confirmed jaguar presence but harboring less than 230 km^2^ of habitat in good condition were classified as ‘small fragments with jaguar presence’ (Fig. 6).

Areas of suitable habitat but without jaguar records, were categorized considering their proximity to an occupied area of the JCU or PJCU and the size of the habitat fragment. A fragment of suitable habitat at <15 km of a JCU or PJCU was categorized as potential expansion area of these units. Isolated fragments of continuous habitat in good condition (medium or high suitability) larger than 230 km^2^ were categorized as potential future core areas (Fig. 6).

### Evaluating the potential connectivity among JCUs

To determine the potential connectivity of jaguar populations among all the JCUs and PJCUs, we used a least-cost functional connectivity model^44^,^64^. We created a resistance to movement surface, which was calculated as an inverse function of our habitat suitability model^65^. This approach assumes that habitat quality has a direct relationship with facility to movement^65^,^66^. To determine the least cost path we used the Linkage Mapper 0.9^67^. This software uses core habitat areas (JCUs and PJCUs) and raster resistance surfaces to identify and map least-cost linkages between adjacent core areas. Linkage Mapper calculates accumulated costs as it moves away from a core area, and takes into account the distance and direction to create a single composite cost-distance grid.

### Evaluation of threats to jaguars in the JCUs

To identify and rank the main threats to jaguars in every JCU, we developed a questionnaire with a list of the known pressures on jaguars in the AF, and asked for potential additional ones. The questionnaire was responded by 9 experts that are conducting research in the different JCUs. This approach has been used before to identify the threats to jaguars on a continental scale^14^. We asked experts to rank potential threats to jaguars, and requested information about recent cases of jaguar mortality induced by humans in the region as a way to corroborate the ranking of threats for every JCU.

Finally, according to the obtained results on population estimates, isolation of every area and jaguar threats, we propose management actions to mitigate the most important threats to jaguars and improve the chances of the species population growth (Table 2 and Fig. 6).

## Data availability

The datasets used in the analysis and the shape files obtained during the current study (habitat suitability model, shape files of the AJO, APJO and important areas for jaguar conservation) will be available in a public repository.

## Additional Information

**How to cite this article**: Paviolo, A. *et al*. A biodiversity hotspot losing its top predator: The challenge of jaguar conservation in the Atlantic Forest of South America. *Sci. Rep.* **6**, 37147; doi: 10.1038/srep37147 (2016).

**Publisher’s note**: Springer Nature remains neutral with regard to jurisdictional claims in published maps and institutional affiliations.

## Supplementary Material

Supplementary Information

## Figures and Tables

**Table 1:** Information of the camera-trap surveys in the Atlantic Forest including number of stations, effort in camera-trap days, rate of records of jaguars, number of individuals recorded (age and sex), density estimate and its 95% confident interval (95%CI).

Survey	Stations	Trap days	Jaguar records/100 camera trap days	Individuals	Density (95% CI) jaguars/100 km^2^
Morombí PR I	5	918	0.11	1 (1 u)	NE
Morombí PR II	10	930	0.75	4 (1 m, 3 u)	NE
Mbaracayú	25	1844	2.87	8 (3 f, 2 m, 3 u)	1.29* (1.29 to 2.8)
Urugua-í	53	2611	0.11	1 (m)	NE
PN Iguazú	46	2942	0.58	6 (3 f, 2 m, 1 u)	NE
Yabotí	60	2676	0.15	1 (m)	NE
Iguazú-San Jorge	49	2287	1.92	13 (6 f, 4 m, 1 u, 2c)	1.2 (0.56 to 1.89)
Iguazú-Urugua-í	47	2327	2.15	16 (8 f, 8 m)	0.89 (0.58 to 1.24)
PN do Iguaçu I	36	3240	0.21	3 (2 h,1 u)	NE
Green Corridor I	80	5038	1.47	21 (10 f, 7 m, 2 u, 2c)	0.91 (0.61 to 1.22)
PN do Iguaçu II	34	3060	0.75	4 (2 h, 2 m)	NE
Green Corridor II	122	5297	3.51	42 (18 f, 12 m, 1 u, 10c)	1.07 (0.8 to 1.33)
Morro do Diabo	36	1440	5.41	10 (6 f, 4 m)	2.39 (2.31 to 2.57)
Ivinhema	13	1495	2.07	12 (8 f, 3 m, 1 u)	1.66 (0.76 to 2.55)
Vale NR I	30	3032	2.74	8 (4 f, 3 m, 1 u)	2.42 (2.01 to 3.26)
Vale NR II	10	3468	0.09	3 (3 f)	NE
Vale NR III	10	3034	0.07	2 (1 m, 1 u)	NE
Vale NR IV	8	1033	3.1	4 (1 f, 3 m)	NE
Vale NR V	30	1440	1.67	2 (2 m)	NE
Carlos Botelho	16	2170	0.23	4 (1 f, 3 u)	NE
Intervales	14	1497	1.94	4 (2 f, 2 m)	NE
Intervales-PETAR	24	2712	1.25	8 (3 f, 3 m, 2 u)	0.66 (0.29 to 1.17)
Juréia-Itatins	21	2483	0	0	NE
Ilha do Cardoso	8	744	0	0	NE
Serra da Bocaina	26	3054	0	0	NE
Santa Virginia	26	2512	0	0	NE
Serra dos Órgãos I	18	1354	0	0	NE
Serra dos Órgãos II	20	6624	0	0	NE
Serra dos Órgãos III	44	4597	0	0	NE
Serra dos Órgãos IV	48	4788	0	0	NE

Abbreviations: (u) adult jaguar of undetermined sex, (m) adult male, (f) adult female, (c) cub and (NE) not estimated due to scarcity of records. *Estimated with CAPTURE + information of the home range size of three individuals monitored with GPS collars in this area.

The number of stations, the effort, the jaguar record rate and the number of individuals correspond to values of the total sampling effort developed in every survey. The density estimation and its 95% CI correspond to values obtained during the portion of the survey that was used to estimate these parameters.

**Table 2:** Information of the priority areas for jaguar conservation in the Atlantic Forest including name, type, total area, area of jaguar occupancy (AJO), area of potential jaguar occupancy (APJO), percentage of the AJO that was surveyed with camera traps, population estimate of this surveys using population models, main threats to jaguars and management recommendation for jaguar conservation.

Name	Type	Total area (km^2^)	AJO (km^2^)	APJO (km^2^)	% AJO surveyed with camera-traps	Population in surveyed areas	Main threats to jaguar population	Main management recommendation
Green Corridor	JCU Type I	14535	13430	1105	62%	52 (38–64)	Jaguar poaching, Kill of jaguars for conflicts with cattle, Road kills	Reduce all sources of jaguar mortality and poaching of prey. Maintain the connectivity between their two main habitat blocks. Evaluate connectivity with Itaipu and San Rafael PJCUs. Monitor jaguar population.
Upper Parana-Paranapanema	JCU Type I	6724	5343	1380	49%	47 (26–67)	Kill of jaguars for conflicts with cattle, Jaguar poaching, Small and isolated area	Reduce all sources of jaguar mortality and the conflict with livestock owners. Improve connectivity of their three main habitat blocks. Evaluate connectivity with Mbaracayu JCU, Itaipu PJCU and the Pantanal. Monitor jaguar population.
Serra do Mar	JCU Type I	13547	7315	6232	32%	14 (6–24)	Scarcity of prey, Jaguar poaching, Habitat conversion	Reduce all sources of jaguar mortality. Stop poaching of prey and palm harvest. Maintain connectivity among its main habitat blocks. Confirm jaguar presence in the APJO areas and monitor jaguar population. Improve the connectivity with the Serra do Mar Norte JCU.
Mbaracayu	JCU Type II	4086	2643	1443	48%	12 (8–18)	Jaguar poaching, Small and isolated area, Scarcity of preys	Reduce all sources of jaguar mortality and poaching of prey. Reduce habitat loss and maintain connectivity among its main habitat blocks. Evaluate connectivity with Upper Parana-Paranapanema JCU, Itaipu PJCU and the Pantanal. Monitor jaguar population.
Linhares-Sooretama	JCU Type II	503	503	0	79%	10 (9–14)	Small and isolated area, Scarcity of prey, Road kills	Reduce all sources of jaguar mortality and poaching of prey. Evaluate the supplementation of individuals. Monitor jaguar population with emphasis in the genetic diversity.
Rio Doce	JCU Type II	1407	1113	294	0	NA	Small and isolated area, Kill of jaguars for conflicts with cattle, Scarcity of preys	Reduce all sources jaguar mortality and the conflict with livestock owners. Evaluate jaguar population including genetic diversity. Evaluate the supplementation of individuals. Evaluate potential connectivity with Cerrado jaguar population.
Serra do Mar Norte	JCU Type II	3915	1006	2908	3%	NA	Scarcity of prey, Jaguar poaching, Habitat conversion	Reduce all sources jaguar mortality and poaching of prey. Improve habitat connectivity with Serra de Mar JCU and neighbor PJCU. Evaluate jaguar populations including genetic diversity. Evaluate the supplementation of individuals.
San Rafael	PJCU	2941	1958	983	0	NA	Unknown	Evaluate the status of the population. If a jaguar population exists, reduce all sources jaguar mortality. Evaluate its main threats and habitat connectivity with Green Corridor and Mbaracayu JCUs.
Itaipu	PJCU	2258	1460	798	0	NA	Unknown	Evaluate the status of the population. If a jaguar population exists, reduce all sources jaguar mortality. Evaluate its main threats and habitat connectivity with Green Corridor, Upper Parana-Paranapanema and Mbaracayu JCUs
Serra dos Órgãos	PJCU	1578	827	751	47%	NA	Unknown	Evaluate the status of the population. If a jaguar population exists, reduce all sources jaguar mortality, and evaluate its main threats. Evaluate habitat connectivity with Serra do Mar Norte JCUs
Itatiaia	PJCU	1336	1000	336	0	NA	Unknown	Evaluate the status of the population. If a jaguar population exists, reduce all sources jaguar mortality, and evaluate its main threats. Evaluate habitat connectivity with Serra do Mar Norte JCU and Campos do Jordao PJCU
Campos do Jordão	PJCU	539	536	3	0	NA	Unknown	Evaluate the status of the population. If a jaguar population exists, reduce all sources jaguar mortality, and evaluate its main threats. Evaluate habitat connectivity with Serra do Mar Norte JCU and Itatiaia PJCU
Small fragments	Small fragment	877	690	187	0	NA	Very small areas	Areas too small to maintain jaguar populations. Reduce jaguar mortality threats. Evaluate connectivity with a JCU. Studies related with jaguar movements in human dominated matrix are recommended
Total		54,245	37,825	16,420	41%	135 (87 to 187)		
