# Supplementary material for: A biodiversity hotspot losing its top predator: The challenge of jaguar conservation in the Atlantic Forest of South America
Source: Sci Rep. 2016 Nov 16;6:37147. doi: 10.1038/srep37147 (PMC5111070; doi:10.1038/srep37147)
Supplement: Supplementary Information [file srep37147-s1.pdf]

Supplementary material online

A biodiversity hotspot losing its top predator: The challenge of jaguar conservation in the Atlantic Forest of South America

Agustin Paviolo, Carlos De Angelo, Katia Maria. P. M. B. Ferraz, Ronaldo G. Morato, Julia Martinez Pardo, Ana C. Srbek-Araujo, Beatriz de Mello Beisiegel, Fernando Lima, Denis Sana, Marina Xavier da Silva, Myriam C. Velázquez, Laury Cullen, Peter Crawshaw, María Luisa S. P. Jorge, Pedro M. Galetti Jr, Mario S. Di Bitetti, Rogerio Cunha de Paula, Eduardo Eizirik, T. Mitchell Aide, Paula Cruz, Miriam L. L. Perilli, Andiara S. M. C. Souza, Verónica Quiroga, Eduardo Nakano, Fredy Ramírez Pinto, Sixto Fernández, Sebastian Costa, Edsel Amorim Moraes Jr. & Fernando Azevedo.

Figure S1. Location of the camera trap surveys in the Atlantic Forest. References: 1) Morumbí PR I, II; 2) Mbaracayú, 3) Urugua-í, 4) PN Iguazú, Iguazú-San Jorge, 5) Yabotí, 6) Iguazú-Urugua-í, Green Corridor I and II, 7) PN do Iguaçu I and PN do Iguaçu II, 8) Moro do Diabo, 9) Ivinhema, 10) Vale NR I, II, III, IV and V, 11) Carlos Botelho 12) Intervales, 13) Intervales-Petar, 14) Juréia-Itatins, 15) Ilha do Cardoso, 16) Serra da Bocaina,17) Santa Virginia, and 18) Serra dos Órgãos I, II, III and IV. The map was created with ArcGis 10.3 (www.arcgis.com).

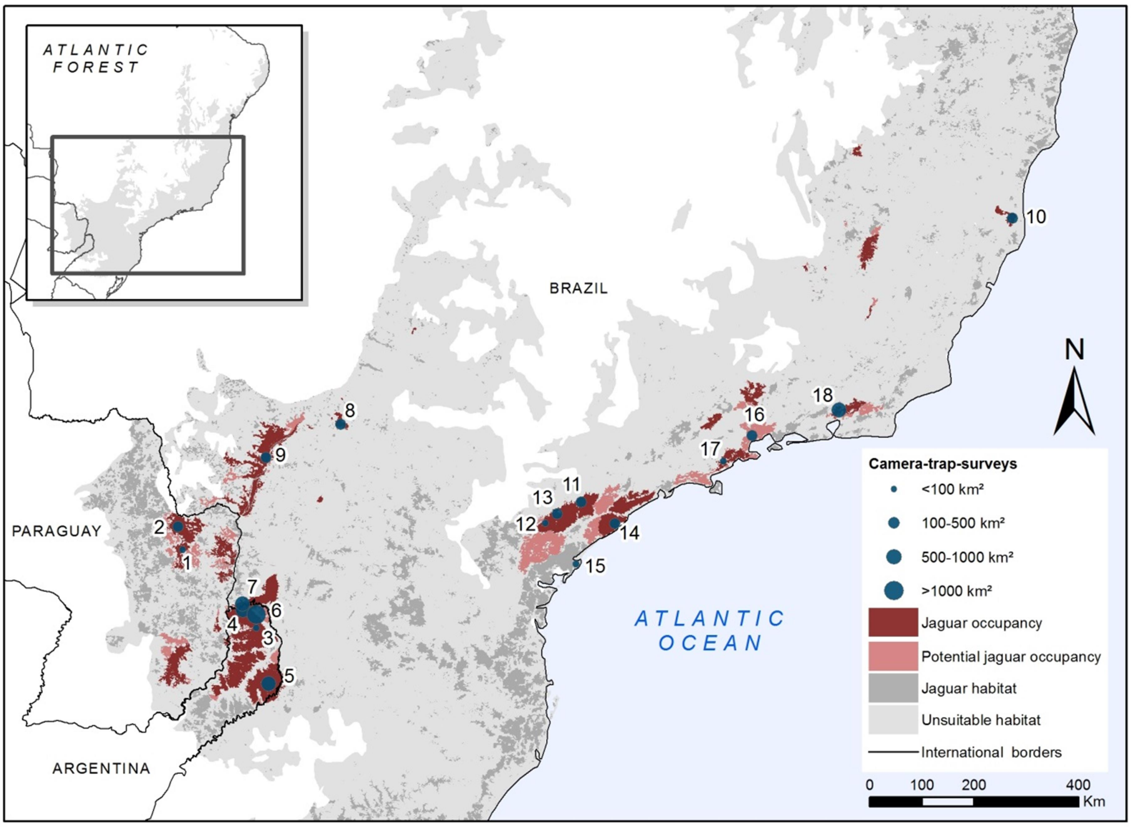

Table S1. Information about the camera traps surveys in the Atlantic Forest. Include are the name of the survey, the region within the Atlantic Forest, the authors, the areas included in the survey, the dates of the survey and the area sampled in km^2 measured as the minimum convex polygon that contain all the camera-traps stations. For more details about field procedures, Cullen et al., 2005; Paviolo et al., 2008; Srbek-Araujo 2013.

Survey	Region of the AF	Author	Areas included in the survey	Dates of survey	Area sampled (km2)
Morombí PR I	East of Paraguay	Velazquez et al.	Morombí PR and an adjacent catlle ranching areas (Paraguay)	4/2010 to 11/2010	8.94
Morombí PR II	East of Paraguay	Velazquez et al.	Morombí PR and an adjacent catlle ranch areas (Paraguay)	6/2011 to 9/2011	76.5
Mbaracayú	East of Paraguay	Velazquez et al.	Mbaracayú Nature Forest Reserve (Paraguay)	12/2009 to 8/2011	188.08
Urugua-í	Green Corridor	Paviolo et al.	Urugua-í PP, Urugua-í WR, private areas of logging companies with pine plantations (Argentina)	5/2003 to 2/2004	81.31
PN Iguazú	Green Corridor	Paviolo et al.	Iguazú NP (Argentina)	4/2004 to 12/2004	204.81
Yabotí	Green Corridor	Paviolo et al.	Esmeralda PP and private areas of logging companies (Argentina)	3/2005 to 12/2005	549.19

Iguazú-San Jorge	Green Corridor	Paviolo et al.	Iguazú NP, San Jorge FR (Argentina) and do Iguaçu NP (Brazil)	4/2006 to 1/2007	569.51
Iguazú-Urugua-í	Green Corridor	Paviolo et al.	Iguazú NP, San Jorge FR, Puerto Peninsula PP, Urugua-í PP, Urugua-í WR, private areas of logging companies with pine plantations (Argentina)	2/2008 to 6/2008	1124.04
PN do Iguaçu I	Green Corridor	Silva et al.	do Iguaçu NP (Brazil)	7/2009-10/2009	556.68
Green Corridor I	Green Corridor	Paviolo et al. and Silva et al.	Iguazú NP, San Jorge FR, Puerto Peninsula PP, Urugua-í PP, Urugua-í WR, private areas of logging companies with pine plantations (Argentina) and do Iguaçu NP (Brazil)	8/2010 to 1/2011	2037.31
PN do Iguaçu II	Green Corridor	Silva et al.	do Iguaçu NP (Brazil)	02/2013-05/2013	533.63
Green Corridor II	Green Corridor	Paviolo et al. and Silva et al.	Iguazú NP, San Jorge FR, Puerto Peninsula PP, Urugua-í PP, Urugua-í WR, Private areas of logging companies with pine plantations (Argentina) and do Iguaçu NP (Brazil)	2/2014 to 7/2014	3212.21
Morro do Diabo	Upper Parana-	Cullen et al.	Morro do Diabo SP (Brazil)	5/2003 to 12/2003	225

	Paranapanema				
Ivinhema	Upper Parana-Paranapanema	Sana et al.	Varzeas do Rio Ivinhema SP (Brazil)	9/2008 to 1/2009	329.67
Vale NR I	Linhares-Sooretama	Srbek-Araujo	Vale Natural Reserve (Brazil)	6/2005 to 6/2006	77.41
Vale NR II	Linhares-Sooretama	Srbek-Araujo	Vale Natural Reserve (Brazil)	6/2006 to 8/2007	169.31
Vale NR III	Linhares-Sooretama	Srbek-Araujo	Vale Natural Reserve (Brazil)	8/2007 to 10/2008	108.51
Vale NR IV	Linhares-Sooretama	Srbek-Araujo	Vale Natural Reserve (Brazil)	6/2009 to 2/2010	122.2
Vale NR V	Linhares-Sooretama	Srbek-Araujo	Vale Natural Reserve (Brazil)	7/2012 to 1/2013	16.25
Carlos Botelho	Serra do Mar	Beisiegel et al.	Carlos Botelho SP (Brazil)	7/2009 to 1/2010	148.63
Intervales	Serra do Mar	Beisiegel et al.	Intervales SP (Brazil)	8/2010 to 2/2011	92.53
Intervales-PETAR	Serra do Mar	Beisiegel et al.	Intervales SP and Alto Ribeira TSP (Brazil)	5/2011 to 10/2011	292.46
Juréia-Itatins	Serra do Mar	Martins and Beisiegel	Jureia-Itatins ES and adjacent areas (Brazil)	4/2012 to 10/2012	258.2
Ilha do Cardoso	Serra do Mar	Nakano-Oliveira	Ilha do Cardoso SP (Brazil)	11/2010 to 2/2011	54.61
Serra da Bocaina	Serra do Mar Norte	Crawshaw et al.	Serra da Bocaina NP, Núcleos Santa Virginia, Cunha and Picinguaba of the Serra do Mar SP and Fazenda Bonito (Brazil)	3/2008 to 6/2009	2875
Santa Virginia	Serra do Mar Norte	Mendonça	Nucleo Santa Virginia of the Serra do Mar SP (Brazil)	4/2013 to 8/2013	31.8
Serra dos Órgãos I	Serra dos Órgãos	Cronemberger	Serra dos Órgãos NP (Brazil)	2010	390
Serra dos Órgãos II	Serra dos Órgãos	Cronemberger	Serra dos Órgãos NP (Brazil)	2011	390

Serra dos Órgãos III	Serra dos Órgãos	Cronemberger	Serra dos Órgãos NP (Brazil)	2012	390
Serra dos Órgãos IV	Serra dos Órgãos	Cronemberger	Serra dos Órgãos NP (Brazil)	2013	390

Abbreviations: (PR) Private Reserve, (PP) Provincial Park, (WR) Wildlife Reserve, (NP) National Park, (FR) Forest Reserve, (SP) State Park, (TSP) State Touristic Park, and (ES) Ecological Station.

References:

Cullen Jr, L., Abreu, K. C., Sana, D. & Nava, A. F. D. Jaguars as landscape detectives for the upper Paraná River corridor, Brazil. *Natureza e Conservação* **3**, 43-58 (2005).

Paviolo, A., De Angelo, C., Di Blanco, Y. & Di Bitetti, M. Jaguar Panthera onca population decline in the upper Parana Atlantic Forest of Argentina and Brazil. *Oryx* **42,** 554-561 (2008)

Srbek-Araujo, A. C. Conservação da Onça pintada (*Panthera onca* Linnaeus, 1758) na Mata Atlântica de Tabuleiro do Espírito Santo. PhD Thesis. Universidad Federal de Minas Gerais (2013).

Table S2. Least cost distance among the all jaguar conservation units (JCUs) and potential jaguar conservation units PJCU. Additionally, we estimated the sum and the minimum connectivity costs for each unit. The sum of the cost of all the corridors represents a general isolation value for this management unit, and the minimum represents the minimum cost distance to the nearest management unit. The values are expressed in thousands of units of relative cost.

**JCU or PJCU**	Campos do Jordao	Green Corridor	Itaipu	Itatiaia	Mbaracayú	Rio Doce	San Rafael	Serra do Mar	Serra do Mar North	Serra dos Orgaos	Upper Parana - Paranapanema	Linhares - Sooretama	**Sum**	**Minimum**
Campos do Jordao		57,172	62,033	1,004	64,642	44,935	63,899	11,693	5,537	10,898	59,876	51,864	**433,552**	**1,004**
Green Corridor	57,172		3,924	56,596	6,534	89,654	3,807	41,187	46,879	55,617	7,199	96,583	**465,155**	**3,807**
Itaipu	62,033	3,924		61,457	444	94,515	8,319	46,048	51,740	60,478	1,601	101,444	**492,002**	**444**
Itatiaia	1,004	56,596	61,457		64,066	42,691	63,323	11,117	4,112	8,973	59,300	49,939	**422,580**	**1,004**
Mbaracayú	64,642	6,534	444	64,066		97,125	7,219	48,658	54,349	63,087	2,108	104,054	**512,286**	**444**
Rio Doce	44,935	89,654	94,515	42,691	97,125		96,381	44,175	37,628	31,536	92,358	30,316	**701,313**	**30,316**
San Rafael	63,899	3,807	8,319	63,323	7,219	96,381		47,914	53,606	62,344	11,699	103,310	**521,821**	**3,807**
Serra do Mar	11,693	41,187	46,048	11,117	48,658	44,175	47,914		1,400	10,138	44,420	51,105	**357,856**	**1,400**
Serra do Mar North	5,537	46,879	51,740	4,112	54,349	37,628	53,606	1,400		3,591	49,583	44,557	**352,983**	**1,400**
Serra dos Orgaos	10,898	55,617	60,478	8,973	63,087	31,536	62,344	10,138	3,591		58,321	38,465	**403,449**	**3,591**
Upper Parana - Paranapanema	59,876	7,199	1,601	59,300	2,108	92,358	11,699	44,420	49,583	58,321		99,287	**485,750**	**1,601**
Linhares - Sooretama	51,864	96,583	101,444	49,939	104,054	30,316	103,310	51,105	44,557	38,465	99,287		**770,924**	**30,316**

Table S3. Variables initially considered as potential predictors to model jaguar distribution in the Atlantic Forest according to previous studies, which included Argentina, Brazil and Paraguay. We selected the better sources of information that were available for the three countries. We applied Pearson's correlation analysis to test the independence between each pair of environmental variables in R (R Core Team, 2013). We excluded variables if their correlation index was highly correlated ($r \geq 0.70$). We prepared all variables in ArcGIS 9.3 at 1 km of spatial resolution and converted to ASCII grid format for the analysis. The final model includes the variables Elevation, Distance from water, Accessibility cost, Forest_1k, Grass_4k and human population density.

Variable	Dataset description	Spatial resolution	Year of data	Source
Elevation	Global elevation data.	30 arc-second	2004	NASA Shuttle Radar Topography Mission
Distance from water	Euclidean distance from the focal pixel to the closest river.	250 m		Rivers from Brazil obtained and updated from IGBE (www.ibge.gov.br/) and from Argentina and Paraguay from De Angelo (2009).
"Riverpc_4k"	Percentage of pixels with rivers calculated by a moving window spatial filter of 4000 m around each focal pixel.	250 m		Rivers from Brazil obtained and updated from IGBE (www.ibge.gov.br/) and from Argentina and Paraguay from De Angelo (2009).
Density of water	Percentage of pixels with water (rivers, lakes, etc) calculated by a moving window spatial filter of 4000 m around each focal pixel.	250 m		Rivers from Brazil obtained and updated from IGBE (www.ibge.gov.br/) and from Argentina and Paraguay from De Angelo (2009). Other water covered areas obtained from the land cover map for 2008 developed

				by Aide et al. (2013),
Accessibility cost	Accessibility cost for humans measured as the hours needed to access the focal cell from the nearest town or city, considering the protected areas as barriers that reduce accessibility.	250 m	2008 - 2014	Developed following the methods and parameters described in De Angelo et al. (2011) but including protected areas (Agostini et al. 2015). Towns and cities obtained from Open Streets Maps (www.openstreetmap.org), roads, rivers and protected areas from Brazil obtained and updated from IGBE (www.ibge.gov.br/) and from Argentina and Paraguay from De Angelo (2009). Land cover obtained from (Aide et al. 2013) and slope calculated from NASA Shuttle Radar Topography Mission.
Land cover	Land cover categories for the Atlantic Forest in 2008 including: forest, water, mixed vegetation, urban, cropland, shrubs, grasslands, bare soil, plantation and marshlands. The selection of the land cover from year 2008 was decided to reduce to the minimum the time frame between the date of the collected records (see Methods S1) and the land cover characteristics.	250 m	2008 - 2009	Land cover map for 2008 developed by Aide et al. (2013), adding marshlands cover from GlobCover Land Cover version v2.3 (http://due.esrin.esa.int/page_globcover.php)
"Forest_1K"	Percentage of forest calculated by a moving window spatial filter of 1000 m around each focal pixel.	250 m	2008	Developed using the forest cover in 2008 estimated by Aide et al. (2013)
"Grass_4K"	Percentage of grasslands and pastures calculated by a moving window spatial filter of 4000 m around	250 m	2008	Developed using the grassland cover in 2008 estimated by Aide et al. (2013)

	each focal pixel.			
"For_marsh_1K"	Percentage of forest and marshlands calculated by a moving window spatial filter of 1000 m around each focal pixel.	250 m	2008-2009	Developed using the forest cover in 2008 estimated by Aide et al. (2013) but including the marshlands mapped by ESA GlobCover 2009 Project
Functional forest amount	Total surface of forest (in km^2) calculated by summing all patches of forest that were accessible from a focal pixel (Jorge et al. 2013) Accessible can be interpreted as connected by other pixel of forest (no barriers or other land covers). Marshlands were included in the same category with forest for this variable calculation.	250 m	2008	Developed using the forest cover in 2008 estimated by Aide et al. (2013)
Human population density	Gridded population of the world v3	2.5 min	2000	Center for International Earth Science Information Network (CIESIN) and Columbia University, and Centro Internacional de Agricultura Tropical – CIAT (http://sedac.ciesin.columbia.edu/data/set/gpw-v3-population-density)
Bioclimatic	Annual mean temperature	30 arc-second	2005	Global Climate Data (www.worldclim.org/bioclim)
Bioclimatic	Mean diurnal range	30 arc-second	2005	Global Climate Data (www.worldclim.org/bioclim)
Bioclimatic	Maximum temperature of warmest month	30 arc-second	2005	Global Climate Data (www.worldclim.org/bioclim)

Bioclimatic	Minimum temperature of coldest month	30 arc-second	2005	Global Climate Data (www.worldclim.org/bioclim)
Bioclimatic	Annual precipitation	30 arc-second	2005	Global Climate Data (www.worldclim.org/bioclim)
Bioclimatic	Precipitation of wettest month	30 arc-second	2005	Global Climate Data (www.worldclim.org/bioclim)
Bioclimatic	Precipitation of driest month	30 arc-second	2005	Global Climate Data (www.worldclim.org/bioclim)

References:

Agostini, I., Pizzio E., De Angelo C. & Di Bitetti M. S. Population status of primates in the Atlantic Forest of Argentina. *J. Primatol.* **36**, 244-258 (2015).

Aide, T. M. et al.. Deforestation and Reforestation of Latin America and the Caribbean (2001–2010). *Biotropica* **45,** 262-271 (2013).

De Angelo, C. El paisaje del Bosque Atlántico del Alto Paraná y sus efectos sobre la distribución y estructura poblacional del jaguar (*Panthera onca*) y el puma (*Puma concolor*). PhD Thesis. Universidad de Buenos Aires, Buenos Aires, Argentina (2009).

De Angelo, C., Paviolo, A. & Di Bitetti, M. Differential impact of landscape transformation on pumas (*Puma concolor*) and jaguars (*Panthera onca*) in the Upper Paraná Atlantic Forest. *Divers. Distrib.* **17,** 422-436 (2011).

Jorge, M. L. S. P., Galetti, M., Ribeiro, M. C. & Ferraz, K. M. P. M. B. Mammal defaunation as surrogate of trophic cascades in a biodiversity hotspot. *Biol. Conserv.* **163,** 49-57 (2013).

R Core Team. R: A language and environment for statistical computing. R Foundation for Statistical Computing, Vienna, Austria. (2013).

Methods S1. Information about the sources and type of data of jaguar presence used to develop and test the habitat suitability model.

The sources of data of jaguar presence that we used to develop the habitat suitability model analysis and to estimate the area of jaguar occupancy in our study came from different sources. Some of the data were collected during a participatory initiative to update the distribution of jaguars in the Upper Parana Atlantic Forest of Paraguay, Brazil and Argentina (De Angelo *et al.* 2011). These data were collected by scientist that developed research activities in the region, and for volunteers of a network composed by common people that search jaguar presence evidences during the development of their daily activities (see De Angelo *et al.* 2011 for more information). The other source of jaguar presence records was an effort developed by the Centro Nacional de Pesquisa y Conservaçao de mamíferos carnivoros de Brasil (CENAP-ICMBio) to compile jaguar records of the Brazilian Atlantic Forest during the development of the National Action Plan for the conservation of the species (Paula *et al.*2011). Jaguar records correspond to camera-trap pictures, locations of collared individuals, poached or road-killed animals, confirmed jaguar attacks to cattle, sightings and jaguar confirmed tracks and feces. The tracks were identified following the methodology developed by De Angelo *et al.* (2010), and the feces identified using molecular techniques to obtain and analyze DNA (Haag *et al.* 2009).

After the conclusion of the jaguar habitat suitability analysis, we collected additional data from the field (n= 107) to use as an independent dataset to further test the model's predictive ability for jaguar occurrence in the AF. These recent data correspond to camera traps pictures, tracks and feces obtained in systematic and unsystematic surveys developed in different regions of the Atlantic Forest.

References:

De Angelo, C. *et al.*. Participatory networks for large-scale monitoring of large carnivores: pumas and jaguars of the Upper Paraná Atlantic Forest. Oryx 45 (4):534-545 (2011).

De Angelo, C., Paviolo, A., & Di Bitetti, M. S. Traditional versus multivariate methods for identifying jaguar, puma, and large canid tracks. The Journal of Wildlife Management, 74(5), 1141-1151 (2010).

Paula, R. C., A. Desbiez & S. M. C. Cavalcanti. Plano de Ação para Conservação da Onça-Pintada no Brasil - Análise de Viabilidade Populacional e Adequabilidade Ambiental. In Série Espécies Amenaçadas (Instituto Chico Mendes de Conservação da Biodiversidade, 2011)

Haag, T., *et al.*. Development and testing of an optimized method for DNA-based identification of jaguar (Panthera onca) and puma (Puma concolor) faecal samples for use in ecological and genetic studies.Genetica, 136(3), 505-512 (2009).

Methods S2. Description of the methodology used to estimate jaguar density with spatially explicit capture-recapture (SECR) models.

Jaguar density was estimated using spatially explicit capture-recapture models (Efford, 2004; Royle et al., 2009). The assumptions of these models are that 1) the study animals have circular home ranges that are constant during the survey, 2) the activity centers are randomly distributed within the study area, 3) the detection probability decrease with distance from the home range center following a detection function, and 4) the population is closed during the survey (Efford, 2004; Royle and Young, 2008).

We defined a priori the extent of the surveyed area as the polygon that includes all the camera traps plus an external buffer of 25 km (see Gopalaswamy et al., 2012). Inside the defined surveyed area we located the potential activity centers uniformly distributed at a distance of 1 km among them. The activity centers that overlay with the area of jaguar occupancy (AJO) were considered as the potential home ranges for the analysis. To run SPACECAP we selected the following model definitions: trap response absent, spatial capture-recapture, half normal detection function, and Bernoulli´s encounter model. We set the MCMC simulations to run 100,000 iterations, discard (burn in) the first 10,000 iterations, assume a thinning value of five and a data augmentation value equal to eight times the number of jaguar individuals recorded in every survey. After every analysis we checked the values of the Geweke test, sample size, model fit, density plot for Psi and N, and the detection function plot to confirm that the results were adequate. If some of these parameters indicated that something was wrong, we ran the models again changing the MCMC simulation parameters until obtaining a model with adequate parameters (for details about all SPACECAP procedures see Gopalaswamy et al., 2012).

SECR models assume a closed population, and to reduce the risk of violation of this assumption, we restricted the density estimation analysis of every survey to a period of between 96 and 120 days, except for the Morro do Diabo and Mbaracayu surveys. For these two surveys because they had a low number of camera traps, we moved the camera traps through different locations during the survey periods to evenly cover these protected areas (Karanth & Nichols, 2002) and used periods of 222 (Morro do Diabo) and 360 days (Mbaracayu). While longer periods increase the chances of violation of the population closure, the isolation of these areas decreases the possibility of jaguar migrations, and the relative long life of jaguars reduced the chances for deaths or recruitment of adult individuals. However, due to the risk of the violation of the closure assumption, we recommend the readers consider the values of density estimates of Mbaracayu and Morro do Diabo with caution.

References:

Efford, M. Density estimation in live-trapping studies. *Oikos* **106,** (3) 598-610 (2004).

Gopalaswamy, A. M., *et al.* Program SPACECAP: software for estimating animal density using spatially explicit capture–recapture models. *Methods Ecol. Evol.* **3,** (6) 1067-1072 (2012).

Karanth, K. U. & Nichols J. D. Monitoring tigers and their prey. A manual for researchers, managers and conservationists in tropical Asia (Centre for Wildlife Studies, 2002).

Royle, J. A., Karanth, K. U., Gopalaswamy, A. M. & Kumar, N. S. Bayesian inference in camera trapping studies for a class of spatial capture-recapture models. *Ecology* **90**, (11) 3233-3244 (2009).

Royle, J. A. & Young, K.V. A hierarchical model for spatial capture-recapture data. *Ecology*, **89**, (8) 2281-2289 (2008).
